# Expression of human RECQL5 in *Saccharomyces cerevisiae* causes transcription defects and transcription-associated genome instability

**DOI:** 10.1007/s00438-024-02152-3

**Published:** 2024-05-26

**Authors:** Juan Lafuente-Barquero, Jesper Q. Svejstrup, Rosa Luna, Andrés Aguilera

**Affiliations:** 1 Centro Andaluz de Biología Molecular y Medicina Regenerativa-CABIMER, Universidad de Sevilla-CSIC-Universidad Pablo de Olavide, 41092 Seville, Spain; 2 Departamento de Genética, Facultad de Biología, Universidad de Sevilla, 41012 Seville, Spain; 3 University of Copenhagen, Copenhagen, Denmark; 4 Francis Crick Institute, London, UK

**Keywords:** RECQL5 helicase, Transcription, Genome instability, Recombination

## Abstract

RECQL5 is a member of the conserved RecQ family of DNA helicases involved in the maintenance of genome stability that is specifically found in higher eukaryotes and associates with the elongating RNA polymerase II. To expand our understanding of its function we expressed human RECQL5 in the yeast *Saccharomyces cerevisiae,* which does not have a *RECQL5* ortholog. We found that *RECQL5* expression leads to cell growth inhibition, increased genotoxic sensitivity and transcription-associated hyperrecombination. Chromatin immunoprecipitation and transcriptomic analysis of yeast cells expressing human RECQL5 shows that this is recruited to transcribed genes and although it causes only a weak impact on gene expression, in particular at G + C-rich genes, it leads to a transcription termination defect detected as readthrough transcription. The data indicate that the interaction between RNAPII and RECQL5 is conserved from yeast to humans. Unexpectedly, however, the RECQL5-ID mutant, previously shown to have reduced the association with RNAPII in vitro, associates with the transcribing polymerase in cells. As a result, expression of RECQL5-ID leads to similar although weaker phenotypes than wild-type RECQL5 that could be transcription-mediated. Altogether, the data suggests that RECQL5 has the intrinsic ability to function in transcription-dependent and independent genome dynamics in *S. cerevisiae*.

**Supplementary Information:**

The online version contains supplementary material available at 10.1007/s00438-024-02152-3.

## Introduction

Helicases are proteins that participate in different and relevant aspects of DNA and RNA metabolism. The RECQ proteins constitute a family of highly conserved DNA helicases from bacteria to humans that are involved in the maintenance of genome integrity with roles in DNA replication, transcription, chromatin remodeling and DNA repair (Croteau et al 2014). Bacterial cells have a single RECQ gene (Bernstein and Keck 2003), unicellular eukaryotes have RecQ homologs, Sgs1 and Hrq1 in *Saccharomyces cerevisiae* and Rqh1 in *Schizosaccharomyces pombe* (Ahmad and Stewart 2005; Groocock et al 2012; Watt et al 1996)*,* while the number of RecQ helicases rises with the complexity of the genome, thus plants and humans have several RecQ helicases with different specialized roles (Das et al 2021; Dorn and Puchta 2019). The human genome encodes five helicases of the RecQ family: RecQ1, BLM (RecQ2), WRN (RecQ3), RecQ4 and RECQL5. Mutations in *BLM*, *WRN* and *RECQ4* genes cause the hereditary Bloom, Werner and Rothmund-Thomson syndromes, respectively, which are associated with a stronger predisposition to cancer and premature aging (Ellis et al 1995; Kitao et al 1999; Yu et al 1996). Despite no human disease being associated with *RECQL5* mutations so far, evidence supports a role for this helicase in the maintenance of genome stability. RECQL5-deficient human cells show high levels of sister chromatid exchange and knockout mice present increased cancer susceptibility (Hu et al 2007). Interestingly, RECQL5 is the only helicase that has been shown to interact with RNA polymerase II (RNAPII) (Aygun et al 2008). RECQL5 negatively regulates transcription elongation, and loss of this helicase results in an increase of RNAPII pausing, stalling and transcription-associated genome instability, especially at common fragile sites (Aygun et al 2009; Kassube et al 2013; Saponaro et al 2014).

RECQL5 functions have been related to the prevention of conflicts between replication and transcription machineries in human cells and in the response to replicative stress (Chappidi et al 2020; Li et al 2015; Popuri et al 2012; Urban et al 2016). It has been shown that this helicase removes Rad51 filaments and facilitates the access of nucleases for the processing of stalled forks to trigger DNA repair (Di Marco et al 2017; Hu et al. 2007). Thus, apart from the role of RECQL5 in transcription, a putative role in replication or DNA repair is yet unclear (Ahamad et al 2021; Hamadeh and Lansdorp 2020).

Studies using *S. cerevisiae* and *Schizosaccharomyces pombe* have contributed to the understanding of the role of *BLM* and its orthologues in multicellular organisms (Ashton and Hickson 2010). *SGS1* is required for maintenance of genome stability and the suppression of illegitimate recombination and is considered the homologue of *BLM* and *WRN* genes (Watt et al. 1995, 1996). In addition, expression of human helicases BLM and WRN can each partially complement yeast *sgs1* null mutants (Yamagata et al 1998). Given the potential of model organisms for the analysis of DNA metabolic processes, we used *S. cerevisiae* to further explore the biological roles of human *RECQL5*, provided that this yeast does not have a recognized *RECQL5* ortholog. We expressed *RECQL5* in yeast and analyzed its impact on transcription and genome instability. We show that *RECQL5* expression leads to cell growth inhibition, increased genotoxic sensitivity and transcription-associated-hyperrecombination. Human RECQL5 interacts physically with yeast RNAPII, is recruited to active chromatin regions along the yeast genome, and causes defective transcription termination detected as readthrough transcription. These results together suggest that RECQL5 may have diverse intrinsic functions in transcription and DNA metabolism and these functions are provided in yeast by other helicases.

## Materials and methods

### Yeast strains, plasmids and other reagents

Strains, plasmids, primers, antibodies, and genotoxic agents used in this study are listed in Supplementary Table 1.

### Yeast cultures

Yeast cells were grown at 30 °C. Media used in this study: YPAD (1% yeast extract, 2% bacto-peptone, 2% glucose, and 20 mg/ml adenine), synthetic defined (SD) (0.17% yeast nitrogen base without amino acids, 0.5% ammonium sulphate, supplemented with amino acids), synthetic complete (SC) (SD with 2% glucose, 2% galactose or 2% raffinose). Solid media were prepared by adding 2% agar. Genotoxic agents were added at the appropriate concentration to molten selective SC medium and poured into petri dish plates to get solidified.

### Western blot

Western blots were performed on proteins extracted with 10% TCA following standard procedures (Kushnirov 2000). Samples were loaded on acrylamide gels, migrated in an SDS-containing buffer, transferred to nitrocellulose membranes and hybridized with anti-FLAG (1:20,000, F3165, Sigma-Aldrich) or anti-Actin (1:2000, ab8224, Abcam) primary antibodies and anti-mouse HRP-conjugated secondary antibody (Mouse: 1:6000, A4416, Sigma; Rabbit: 1:10,000, A6154, Sigma).

### Detection of Rad52 and Rad51 foci

Spontaneous Rad52-YFP foci from mid-log cells carrying plasmid pWJ1344 were visualized and counted by fluorescence microscopy as previously described ((Lisby et al 2001) using a Leica DC 350F microscope. More than 200 S/G2 cells were inspected for each experimental replica. Experiments were performed with yeast cultures of transformants carrying plasmids with *tet::RECQL5* or *GAL::RECQL5* grown in selective SC medium with 2% glucose or SC with 2% galactose, respectively. To study the effect of RNase H1 (RNH1) overexpression on Rad52 foci formation cells were also transformed with a plasmid carrying RNH1 under the control of tet promoter (pCM184RNH1) (Santos-Pereira et al 2013). For Rad51-YFP foci the strain ML149-8A was used (Torres-Rosell et al 2007). The average and SEM of at least three independent transformants was plotted.

### Analysis of recombination frequencies

Recombination frequencies were determined as described (Gómez-González et al 2011b). Chromosomal *leu2-k::ADE2-URA3::leu2-k* system and plasmids pRS314L, pRS314LY, and pSch204, pRS314GL-*lacZ* were used to determine recombination frequencies. Briefly, transformants with indicated plasmids were grown for 3–4 days (until similar colony size) in the appropriate selective media SC with 2% glucose (for plasmids with *tet::RECQL5* or *tet::RECQL5-ID*) or 2% galactose supplemented with 0.05% glucose (for plasmids with *GAL::RECQL5* or *GAL::RECQL5-ID*) when applicable. In the case of pSch204, pRS314GL-*lacZ* plasmid-systems yeast cells expressing *tet::RECQL5* or *tet:RECQL5-ID* were grown in selective SC with 2% glucose or in selective SC with 2% galactose to determine recombination frequencies in low or high transcription conditions, respectively. Recombinants were obtained by plating appropriate dilutions and selected as Leu + colonies for the plasmids containing *LEU2* truncated repeat systems. Recombination analyses for the chromosomal leu2-k::ADE2-URA3::leu2-k system (Lk-AU) were performed using 6 to 12 independent colonies grown in synthetic complete medium SC with appropriate amino acid selection, and recombinants were selected in SC + FOA.

For each strain, the recombination frequencies are given as the average and standard deviation of the median frequency value obtained from fluctuation tests performed in 3–4 different transformants using 6 independent colonies per transformant. These transformants come from one or two independent transformations; in any case, they are different and the analysis of 6 colonies of each one provides enough sample to perform the test according to the standard methodology (Gómez-González et al. 2011b).

### Co-immunoprecipitation assays

Strains carrying plasmids with *GAL::RECQL5*, *GAL::RECQL5-ID*, or the empty vector (pYES) were grown in selective SC medium with 2% raffinose to mid-log phase and then 2% galactose was added for 4 h to induce protein expression. For protein complex precipitation, 50 ml of exponentially growing cells were harvested and washed. 100 µL of glass beads and 166 µL of Lysis Buffer (50 mM Tris HCl pH 7.5, 100 mM NaCl, 1.5 mM MgCl_2_, 0.0075% NP40 10%, 100 mM DTT) were added and samples were vortexed in the FastPrep (intensity 5 m/s) 4 × 20″. After separating the beads, samples were centrifuged 10′ at 13,000 rpm. Protein amount in supernatant (crude extract) was quantified using Bradford. Extracts were homogenised to 5 mg/ml, 10 µl were separated for INPUT and the remainder was incubated with 10 µl of a FLAG antibody attached to Dynabeads Protein A (Invitrogen) for 3 h at 4 °C. Magnetic beads were centrifuged, washed 3 × with ice-cold PBS 1 × and resuspended in 40 µl Laemmly Buffer 1x. Samples were boiled 5′ before loading the gel.

### Chromatin immunoprecipitation experiments

Recruitment of *RECQL5-FLAG*, *RECQL5-ID-FLAG* and RNAPII to chromatin (*GAL1* locus) was analyzed in exponentially growing cells carrying plasmids for ectopic expression of helicase Flag-tagged proteins under the control of tet promoter (*tet::RECQL5* or *tet::RECQL5-ID*) or the empty vector in the appropriate synthetic medium. Yeasts cultures grown were grown in selective SC medium with 2% raffinose to mid-log phase and then 2% glucose or 2% of galactose was added for 4 h to analyze protein recruitment at *GAL1* gene under repressed or active conditions, respectively. ChIP analyses were performed as previously described (Gómez-González et al 2011a) using the monoclonal antibodies anti-FLAG-M2 and anti-Rpb1-CTD antibody 8WG16 (Berkeley Antibody Company, Richmond, CA, USA) and Dynabeads protein A (Invitrogen). For qPCR, the intergenic region at positions 9716–9863 of chromosome V was used as a negative control. Average and SEM of two independent experiments are shown.

### RNA extraction and northern blot

Strains carrying plasmids with *GAL::RECQL5*, *GAL::RECQL5-ID*, or the empty vector (pYES) were grown selective in SC medium with 2% raffinose to mid-log phase and then 2% galactose was added to induce transcription from the *GAL1* promoter. Samples were taken at different times and RNA extraction was performed by acid phenol following standard procedures. Northern blot of kinetics was carried out as previously described (Chávez and Aguilera 1997). RNA was separated by agarose electrophoresis in a gel containing MOPS 1 × and 2% formaldehyde and transferred to Hybond-N nitrocellulose membranes (GE Healthcare). *GAL1* and *GAL10* probes were amplified by PCR using primers detailed in Supplementary Table 1. Probes were ^32^P-labelled using Klenow polymerase (Roche) and ^32^P-dCTP. Signal was acquired using a FLA-5100 Imager Fluorescence Analyzer (Fujifilm) and quantified with MultiGauge 2.0 analysis software (Science Lab). Signal was measured as arbitrarily units (a.u.) and normalized to control condition.

### Microarray gene expression analysis

Strains carrying plasmids with *tet::RECQL5*, or the empty vector were grown in selective SC medium with 2% glucose and without doxycycline (tet ON). Total RNA was isolated from 50 ml of mid-log growing cultures using the RNeasy Midi kit (Qiagen). Global expression analyses were performed with Affymetrix platform GeneChip Yeast Genome 2.0 Array. The relative RNA levels for all yeast genes were determined using an Affymetrix microarray scanner. For the RECQL5-expressing cells (*tet::RECQL5)*, microarray analysis was conducted in triplicate and the values presented represent the average of these three determinations, control not expressing RECQL5 was performed only once and standardized with published data of experiments performed in the same conditions GSE22644 (Tu et al 2011) (Pearson’s R correlation coefficient of 0.914). A total of 7.5 µg of RNA were used for cDNA synthesis, labelling and microarray hybridization, performed by the CABIMER’s Genomics Unit according to the manufacturer’s instructions. Microarray data were normalized by RMA (robust microarray average) and statistically analyzed by LIMMA (Linear Models for Microarray Analysis) comparing the RECQL5-expressing cells profile with its isogenic wild-type strain. Genes with expression levels below 600 (the median expression value of non-expressed meiotic genes in these experiments) were removed from the analysis to reduce false positive. Genes showing at least a 1.5-fold expression change with a FDR < 0.1 were considered as altered. GO analyses were performed using the GO Term Finder tool from the *Saccharomyces* Genome Database (http://www.yeastgenome.org). A p-value < 0.01 was established to consider GO terms as significantly enriched. The data generated are available under Gene Expression Omnibus accession number (GSE180112).

### ChIP-chip experiments

ChIP-chip analysis were carried out in cells expressing RECQL5-FLAG tagged protein under the tet promoter (*tet::RECQL5*). Yeast cultures were grown to mid-log phase in selective SC medium containing 2% glucose and without doxycycline. *S. cerevisiae* oligonucleotide tiling microarrays were provided by Affymetrix (GeneChip *S. cerevisiae* Tiling 1.0R array). The high-density oligonucleotide arrays used allows the analysis of yeast chromosomes at a 300-bp resolution, each of the 300-bp region being covered by at least 60 probes. ChIP-chip of asynchronously growing cells was carried out as described (Bermejo et al 2009; Gómez-González et al. 2011a). For immunoprecipitation with RECQL5-FLAG, cells growing in SC-T were harvested at mid-log phase. 1.5 × 10^7^ cells were disrupted by multi-beads shocker (MB400U, Yasui Kikai, Japan), which maintained cells precisely at lower than 4 °C during disruption. Anti-FLAG antibody M2 (Sigma-Aldrich) was used for ChIP. ChIP DNA was purified and amplified by random priming using a WGA2 kit (Sigma- Aldrich) and following the manufacturer’s procedure. A total of 4 μg of amplified DNA was digested with DNaseI to a mean size of 100 bp and the purified DNA fragments were end-labelled with biotin-N6-ddATP23. The ChIP-chip data can be accessed at Gene Expression Omnibus (GSE180107). Annotation of RECQL5-enriched regions were done using in-house scripts with the latest version of the SGD_features.tab (based on Genome Version R62-2-1) hosted by SGD (SGD Project. http://www.yeastgenome.org/download-data/ [April 2021]).

### Statistical analysis of genome-wide data

Microarray expression data were normalized by RMA (robust microarray average) and statistically analyzed by LIMMA (linear models for microarray analysis), comparing the mutant expression profile with its isogenic wild-type strain. The genes showing at least a 1.5-fold expression change with a p-value < 0.01 with a false discovery rate (FDR) corrections were considered as significantly altered.

ChIP–chip data were analyzed using the Tiling Array suite (TAS) software from Affymetrix. For each probe position, TAS produces the signal and the change p-value, taking into account the probes localized within a given bandwidth around the inspected probe. Protein chromosomal distribution was then analyzed by detecting binding clusters, which were defined as ranges within the chromosome respecting the following conditions: estimated signal (IP/SUP-binding ratio) positive in the whole range, p-value < 0.01, minimum run of 100 bp, and maximum gap of 250 bp.

The results were visualized with the UCSC Genome Browser, developed and maintained by the Genome Bioinformatics Group (Center for Biomolecular Science and Engineering at the University of California at Santa Cruz; http://genome.ucsc.edu/). Mapping of binding clusters into Stanford Genome Database genomic features (www.yeastgenome.org) was performed using in-house developed Perl scripts. To visualize the distribution of binding sites along ORFs, these were divided into equivalent segments from the start and end coordinates as previously described (Gómez-González et al. 2011a; Santos-Pereira et al. 2013).

## Results

### RECQL5 expression hypersensitizes yeast cells to replicative stress and inhibits growth

**Fig. 1:**
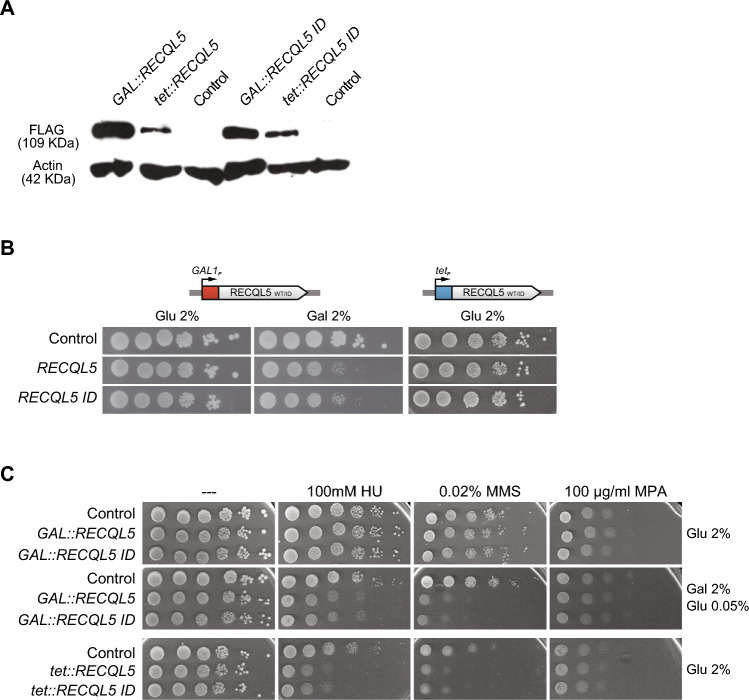
Expression of human helicase RECQL5 in *S. cerevisiae* increases sensitivity to genotoxic agents. **A** Expression levels of the wild-type RECQL5 and the RECQL5-ID versions placed under the control of *GAL1* or tet promoters. Western blot analysis using the FLAG antibody to detect the tagged RECQL5 protein levels. Yeasts carrying plasmids with *GAL::RECQL5*, *GAL:RECQL5-ID*, or the control (empty pYES3) were grown in selective SC medium with 2% raffinose to mid-log phase and then 2% galactose was added for 4–6 h to induce protein expression from *GAL1* promoter. Strains carrying plasmids with *tet::RECQL5*, *tet::RECQL5-ID* or the control (empty pCM184) were grown in selective SC medium with 2% glucose and without doxycycline (tet ON). **B** Effect of RECQL5 expression in WT cells (W303-1AR5). Ten-fold serial dilutions of WT (W303-1AR5) transformed with plasmids containing *GAL::RECQL5*, *GAL:RECQL5-ID*, *tet::RECQL5, tet:RECQL5-ID* constructs or control (respective empty plasmid) and plated on selective SC medium supplemented with the indicated carbon source are shown. Cells were diluted in sterile distilled water. Photographs were taken after 2–3 days of growth at 30 °C for transformants expressing the human helicase under the control of *tet* or *GAL1* promoter respectively. **C** Analysis of sensitivity RECQL5-expressing yeast to genotoxic agents: hydroxyurea (HU), methylmethanesulfonate (MMS), or the transcription elongation inhibitor mycophenolic acid (MPA), ten-fold serial dilutions of WT (W303-1AR5) transformed with the respective plasmids and plated on minimal selective medium supplemented with the indicated carbon source are shown. Other details as in panel B (**B**)

To explore how expression of *RECQL5* affects yeast physiology we used a human cDNA, corresponding to the wild-type *RECQL5*, and a mutated version, called *RECQL5-ID*, deficient in the RNAPII-interacting domain previously described and used as control (Aygun et al. 2009). We cloned each cDNA under the control of either the Tet-off promoter, repressible by doxycycline, or the strong *GAL1* promoter, inducible by galactose in centromeric and multicopy plasmids, respectively. Expression of human *RECQL5* and *RECQL5-ID* in yeast transformed with these plasmids was achieved from both constructs as determined by western blot, the expression levels being higher for the *GAL1p* than for the *tet*-based constructs (Fig. 1A). Interestingly, we observed a growth inhibition phenotype associated with higher levels of RECQL5 overexpression under *GAL1* promoter. This phenotype was alleviated in strains carrying *GAL::RECQL5* versions cultured in 2% galactose-containing medium with the presence of small amounts of glucose, that results in lower levels of overexpression (Fig. 1C; Supplementary Fig. 1) Consistently, yeast cells expressing *tet::RECQL5* constructs did not show growth impairment (Fig. 1B).

Since RECQL5 functions in transcription and in the maintenance of genome integrity in mammalian cells (Croteau et al. 2014), we wondered whether its expression had any effect in the DNA damage response (DDR) in yeast. We assayed first whether it affected the sensitivity of wild-type yeast cells to genotoxic agents like hydroxyurea (HU), which inhibits replication by dNTP depletion; methyl-methane-sulfonate (MMS), an alkylating agent that causes double-strand break (DSBs) among other lesions; and mycophenolic acid (MPA), a transcription elongation inhibitor. As can be seen (Fig. 1C), overexpression of both wild-type *RECQL5* and mutant *RECQL5-ID* indistinctively conferred sensitivity to HU and MMS, whereas it did not affect MPA sensitivity. These results suggest that expression of human RECQL5 in yeast interferes with DNA repair, but, if any, poorly with transcription elongation.

### RECQL5 expression leads to transcription-associated genome instability in yeast

**Fig. 2:**
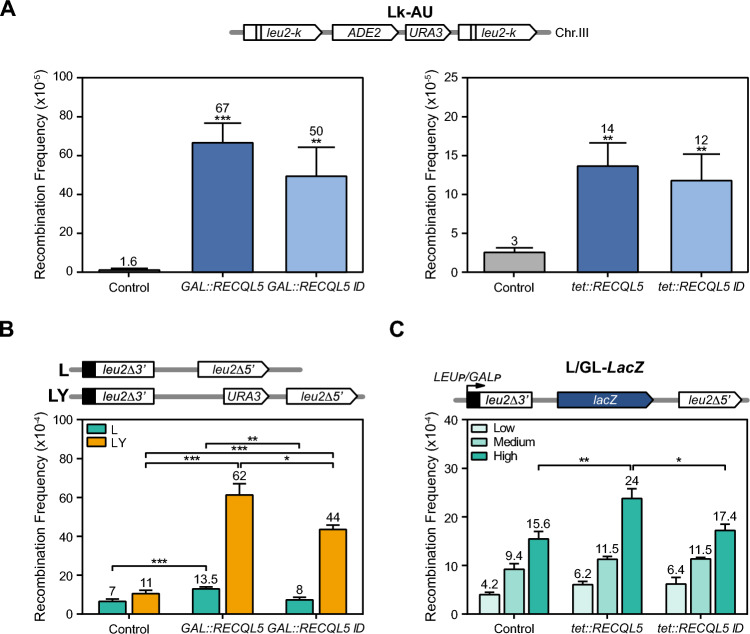
Genome instability in RECQL5-expressing cells. **A** Recombination analysis in the WT strain (AYW3-1B) with the chromosomal Lk-AU system and transformed with the indicated plasmids. Cells were grown for 3–4 days in selective media SC with 2% glucose (for *tet::RECQL5* and *tet::RECQL5-ID*) or 2% galactose supplemented with 0.05% glucose (for *GAL::RECQL5* and *GAL::RECQL5-ID*). Control refers to the respective empty plasmid. **B** Recombination analysis of WT cells (W303-1AR5) carrying L and LY plasmid system and expressing *GAL::RECQL5* or *GAL:RECQL5-ID* constructs. Other details as in panel A. **C** Recombination analysis of WT cells (W303-1AR5) expressing *tet::RECQL5* or *tet::RECQL5-ID* constructs in the GL-*lac*Z system under the control of the inducible *GAL1* promoter in glucose (low transcription-GAL1p OFF), in galactose (High transcription-GAL1p-ON) or the L-*lac*Z system expressed under the control of the constitutive *LEU2* promoter in glucose (Medium transcription-LEU2p-ON). Average and SEM of at least three different fluctuation tests from six independent colonies each one are shown. *, p ≤ 0.05; **, p ≤ 0.01; ***, p ≤ 0.005; (Student’s t-test)

The yeast sensitivity to genotoxic agents caused by RECQL5 expression suggests an interference with the DNA repair or replication processes that could potentially affect genome stability. This prompted us to assay whether RECQL5 expression leads to genome instability associated with transcription. We first analyzed whether RECQL5 expression generated a hyper-recombination phenotype in wild-type yeast. For this, we used different *leu2* direct-repeat constructs to measure single strand annealing (SSA), a highly efficient mechanism of DSB repair independent of Rad51, which serves to infer whether DNA breaks are stimulated. We assayed SSA recombination in the chromosomal Lk-AU system which is based on two heteroallelic 2.16-kb *leu2* repeats flanking the *URA3* and *ADE2* genes (Aguilera and Klein 1989). Recombination between the two *leu2* repeats leading to *URA3* deletions was scored as the frequency of *Ura*^−^ cells, detected as FOA-resistant colonies. A clear increase of the recombination frequencies was observed upon expression of both wild-type RECQL5 and RECQL5-ID respect to the control cells, the values being slightly lower upon expression of the mutant allele. The levels of hyper-recombination were dependent on the RECQL5 expression levels (Fig. 2A), proportional to the strength of the promoter used, whether *GAL1* or *tet* promoters.

**Fig. 3:**
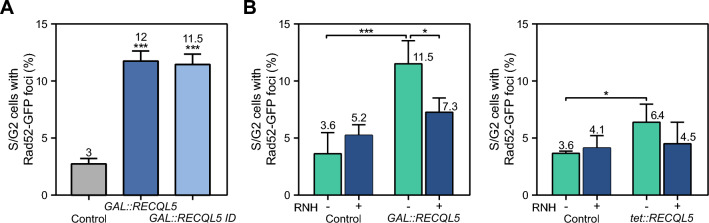
Expression of human helicase RECQL5 in *S. cerevisiae* leads to R-loop dependent genome instability. **A** Spontaneous Rad52-YFP foci formation in WT (W303-1AR5) expressing *GAL::RECQL5* or *GAL::RECQL5-ID*. **B** Effect of RNase H1 (RNH1) overexpression on Rad52 foci formation in cells carrying plasmids with *GAL::RECQL5* or *tet::RECQL5* constructs and transformed with either the plasmid pCM184RNH1 (tet::RNH1) or the empty vector pCM184. Control refers to the respective empty plasmid without RECL5. Average of at least three independent experiments are shown (*, p < 0.05; **, p < 0.0005). Yeast cultures were grown in selective SC medium (*tet::RECQL5*) or switched from S-Raffinose to SC with 2% galactose until exponential (4–6 h) for *GAL::RECQL5*

For a better association of hyper-recombination with transcription levels we next analyzed SSA recombination in the plasmid-born systems L and LY. These systems are based on the same direct-repeats (600-bp internal fragments of the *LEU2* gene sharing 300 bp of homology) that are transcribed from the *LEU2* promoter, but they differ in the length of the transcribed intervening sequence (31 bp for L, and 5.57 kb for LY) (Prado and Aguilera 1995) (Fig. 2B). Expression of both *RECQL5* and *RECQL5-ID* increased SSA recombination in the LY system (5.6-4 fold), whereas in the L system the increase was weak (twofold for RECQL5) or non-existing (RECQL5-ID). This result indicates that increase of SSA events is significant in the systems, like LY, generating a long transcript. Then, we determined the effect of RECQL5 and RECQL5-ID expression in the L-lacZ and GL-lacZ plasmid recombination systems that contain the GC-rich sequence *lacZ* between 0.6-kb *leu2* direct-repeats transcribed from different promoters, *LEU2* promoter (L-lacZ system) or *GAL1* promoter (GL-lacZ system) (Fig. 2C). We have previously used these systems to report the transcription-associated recombination (TAR) phenotype of different mRNP biogenesis mutants (Chávez and Aguilera 1997; González-Aguilera et al 2008; Piruat and Aguilera 1998). Similar increases were obtained in the L-lacZ and GL-lacZ systems that also generated a long transcript (> 4 kb). Importantly, hyper-recombination was significantly higher in the GL-lacZ system, compared to the L-lacZ, consistent with the higher transcription (Fig. 2C). The results therefore, clearly show that *RECQL5* expression increases recombination in a transcription-dependent manner, a phenotype also observed for the mutant RECQL5-ID, although to a lesser extent. The high SSA levels suggest that *RECQL5* expression leads to the accumulation of recombinogenic DSBs. To assess this more directly we tested Rad52-YFP foci accumulation in yeast expressing RECQL5 and RECQL5-ID. A fourfold increase in Rad52-YFP foci was detected in both cases (Fig. 3A), consistent with an increase in recombinogenic DSBs. However, Rad52 foci could also accumulate as a consequence of a lower DSB repair efficiency. To test this possibility, we analyzed accumulation Rad51 foci, provided that Rad51 acts in the HR reaction after Rad52 by forming nucleoprotein filaments. Interestingly Rad51 foci were clearly reduced compared with the wild-type control (Supplementary Fig. 2), suggesting that instability generated by RECQL5 expression in yeast may be produced by both the occurrence of DNA breaks and interference with the DNA repair process.

Taking into account the roles of RECQL5 in transcription and replication processes and the impact of R-loops as a source of transcription-replication conflicts and genome instability, we asked whether the increase in DNA breaks associated to RECQL5 expression was dependent on R-loops. We assayed whether Rad52 foci accumulation was suppressed by RNase H1 (RNH1), an enzyme that degrades the RNA strand of a DNA-RNA hybrid (Cerritelli and Crouch 2009). Since there were no consistent differences in the phenotype when expressing the wild-type of the ID mutant version of RECQL5, we only assayed the effect of the wild-type RECQL5 expression from the *GAL1* or *tet* promoters (Fig. 3B). A reduction of Rad52 foci formation by RNH1 expression was observed in *GAL::RECQL5* and *tet::RECQL5* expressing cells suggesting that RECQL5 expression leads to R-loop-dependent genome instability.

### Human RECQL5 interacts in vivo with yeast RNAPII impairing transcription

**Fig. 4:**
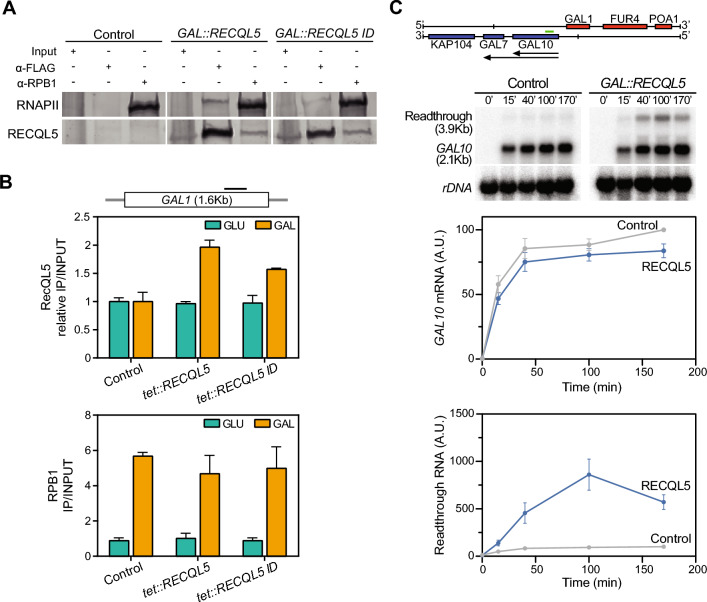
Interaction of the human helicase RECQL5 with active chromatin and its impact on transcription in *S. cerevisiae.* **A** RNAPII protein interaction with either RECQL5 or RECQL5-ID FLAG-tagged versions detected by co-immunoprecipitation with anti-Rpb1 (8WG16) or anti anti-FLAG antibodies from whole cell extracts of the yeast carrying the empty vector (control) or the indicated *GAL::RECQL5* constructs. Yeasts carrying plasmids with *GAL::RECQL5*, *GAL:RECQL5-ID*, or the control (empty pYES) were grown in selective SC medium with 2% raffinose to mid-log phase and then 2% galactose was added for 4 h to induce protein expression. **B** ChIP analysis of RECQL5 FLAG-tagged proteins (upper panel) or RNAPII (lower panel) within the *GAL1* gene in yeasts transformed with the empty plasmid (control) or the indicated *tet::RECQL5* constructs. Assays were performed from yeast cells grown under conditions of active (GAL) or repressed (GLU) transcription for *GAL1* locus. Values represent the relative ratio to empty vector of precipitated DNA (IP) to input DNA (INPUT) for the FLAG antibody, or the ratio of IP to INPUT for the RNAPII antibody. Mean ± SEM of two independent experiments are shown. Schematic diagram of gene is depicted. The black line indicates the region where ChIP-qPCR analyses were performed. **C** Northern blot analysis of *GAL10* mRNAs in WT strain transformed with *GAL::RECQL5* or the empty vector (Control). Graphics represent the corresponding mRNA signal relative to rDNA. Average and SEM of n = 3. Other details as in (**A**)

Since RECQL5 is the unique RecQ helicase that interacts with RNAPII and functions in transcription elongation in human cells (Saponaro et al. 2014), we next asked whether this capability to interact with the transcription machinery and chromatin was conserved in yeast. For this, we did co-immunoprecipitation (Co-IP) assays in cells transformed with plasmids expressing *RECQL5* or *RECQL5-ID* fused to the FLAG epitope, using an anti-FLAG antibody and an antibody against the Rpb1 subunit of RNAPII. As can be seen (Fig. 4A), human RECQL5 immunoprecipitated with yeast Rpb1, implying a physical interaction with the RNAPII transcription machinery. The interaction could be seen regardless of whether we pulled down proteins with RECQL5 or Rpb1 (Fig. 4A). To confirm that this occurred in active chromatin, we performed ChIP-qPCR in the inducible *GAL1* gene. The results clearly showed that when *RECQL5* was expressed from the *tet::RECQL5* construct it was recruited to the *GAL1* gene when transcription was active (galactose) but not when it was repressed (glucose) (Fig. 4B upper panel). Unexpectedly, however, both the wt RECQL5 and mutant RECQL5-ID proteins interacted similarly with yeast chromatin in a transcription-dependent manner. Finally, ChIP analysis indicate that the expression of RECQL5 did not significantly affect the recruitment of RNAPII to the transcribing gene (Fig. 4B lower panel).

To further explore how human RECQL5 could functionally affect yeast RNAPII in *S. cerevisiae* we analyzed the possible genetic interactions between RECQL5 expression and mutations of different RNAPII subunits, *rpb1-1*, *rpb1S751F*, *rpb2-10* and *rpb9∆*, all of which are defective in transcription elongation (Felipe-Abrio et al 2015; Powell and Reines 1996; Strathern et al 2013; Van Mullem et al 2002). We assayed growth of these mutants when they expressed RECQL5. No clear differences in growth were observed in any of the mutants tested when expressing RECQL5 at low (2% Gal + 0.05% Glu) or high levels (2% Gal), although a slight effect in the growth of *rpb1S751F* mutant upon RECQL5 repression conditions (2%Glu) was apparent (Supplementary Fig. 3). Interestingly, this mutation was reported to cause RNAPII slippage as also described for mutants of the elongation factor TFIIS (Strathern et al. 2013). Considering the described competition of RECQL5 with the binding of TFIIS to RNAPII (Kassube et al. 2013) data suggest that expression of human RECQL5 could interfere with the interaction of RNAPII and TFIIS in yeast.

For a more accurate analysis of transcription, we determined the kinetics of activation of the *GAL1, 10 locus* in wild-type cells expressing *GAL::RECQL5* (Fig. 4C). Northern blots of the *GAL10* and *GAL1* genes showed no significant defects of transcription activation upon galactose addition, but interestingly, a larger mRNA resulting from readthrough at termination could be detected (Fig. 4C) (Supplementary Fig. 4). A similar effect was also observed in cells expressing *RECQL5-ID* (Supplementary Fig. 4). Altogether, these data suggest a transcriptional termination defect upon *RECQL5* expression.

### Genome-wide analysis of human RECQL5 expressed in yeast cells

To better understand the impact of *RECQL5* expression on global gene expression, we performed a transcriptomic analysis of cells expressing *tet::RECQL5*. We used the *tet* promoter instead of *GAL1* because it was less toxic and the expression levels were better adjusted to the levels of the transcription machinery. We found that 56 genes were up-regulated (Linear fold-change ≥ 1.5; p-value-FDR ≤ 0.1). They represented a group of long genes (more than 1 kb longer than the genome median) with a GC-content slightly but significantly lower than the median value of the genome. Moreover, 89 genes were down-regulated in RECQL5-expressing cells (Supplementary Table 2). These downregulated genes were highly expressed in normal conditions, consistent with the observation that RECQL5 binds to actively transcribed genes, and, thus, control them. Interestingly, downregulated genes were shorter and with a higher GC content than the median of the genome (Supplementary Fig. 5). In general, up-regulated genes were involved in RNA biogenesis and rRNA metabolism (GO:0042254, 17 of 56 genes, p-value 2.73E-5, and GO:0016072, 13 of 56 genes, p-value 0.0046), down-regulated transcripts were enriched in functions of general amino acid metabolism (GO:0006520, 30 out of 89 genes, p-value 1.25E-19) (Supplementary Table 3). In addition, a number of DDR genes were deregulated under RECQL5 expression (Supplementary Table 2, highlighted in red).

**Fig. 5:**
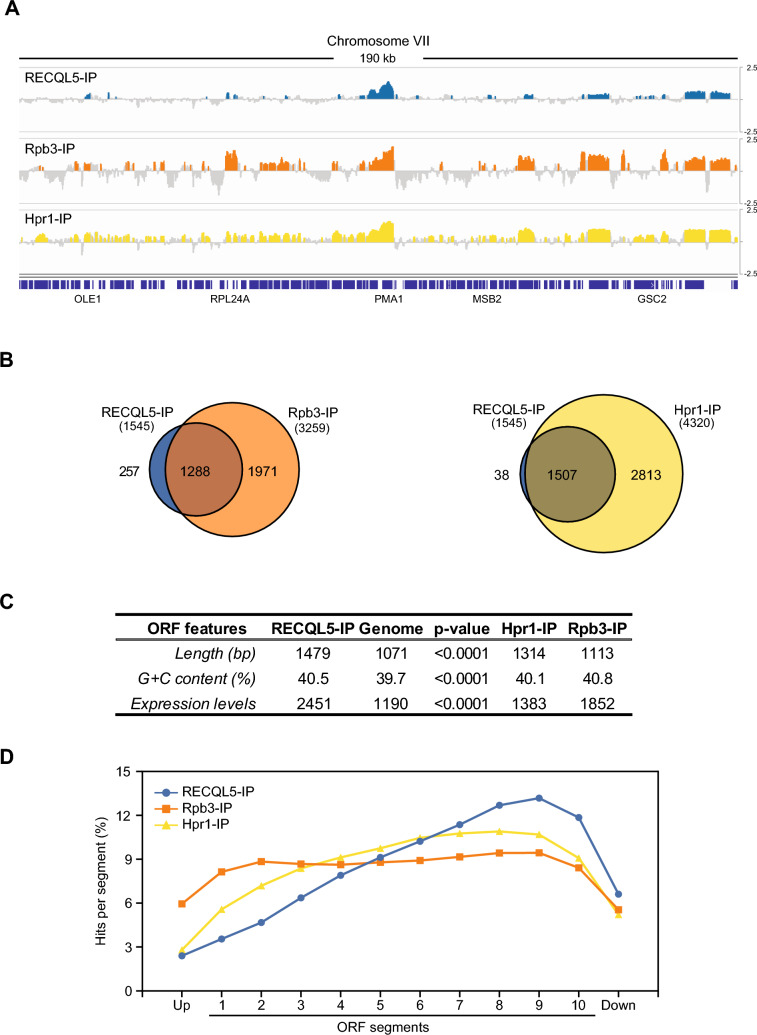
Genome-wide analysis of recruitment of human RECQL5 expressed in *S. cerevisiae.* **A** Recruitment pattern of RECQL5, Rpb3 and Hpr1 to ORFs. ChIP-chip analysis of RECQL5-FLAG of cells expressing *tet::RECQL5* and comparison with previously published Rpb3-FLAG and Hpr1-FLAG ChIp-chip data. A genomic fragment from chromosomes VII is shown, with enrichment values represented as signal log2 IP/SUP ratio. Blue (RECQL5-IP), orange (Rpb3-IP) and yellow (Hpr1-IP) histograms (online version) represent the significant binding clusters (p < 0.01, minimum run > 100 bp, maximum gap < 250 bp). Genes and other features are represented according to the *Saccharomyces* Genome Database (SGD) as blue bars and white arrows in relation to the direction of transcription. **B** RECQL5-enriched genes overlap with similarly identified ORFs in Rbp3-IP or Hpr1-IP. **C** Statistical analysis of length, C + G content and expression levels of the RECQL5-enriched genes. Median values for RECQL5-IP, the genome Rpb3-IP and Hpr1-IP plotted. p-value calculated by Mann–Whitney’s U-test. **D** Composite profile of RECQL5 and Hpr1 occupancy detected by ChIP-Chip across the average ORF plotted as RECQL5, Rpb3 or Hpr1 percentage of ChIP clusters per segment (see Materials and Methods)

We extended then our analysis to a genome-wide level and performed ChIP-chip using RECQL5-FLAG tagged protein under the *tet* promoter *(tet::RECQL5)*. Data were subjected to computational analysis to obtain a genomic map distribution (Fig. 5A, Supplementary Table 4) and were compared with previously published Rpb3-HA and Hpr1-Flag ChIP–chip data, the last one used as a control of a cotranscriptional mRNP biogenesis factor (Gómez-González et al. 2011a). Statistical analysis showed that 38.6% of the RECQL5 peaks were at ORFs. We found that 83 and 97.5% of RECQL5-enriched ORFs were also enriched in Rpb3 and Hpr1, respectively (Fig. 5B), strengthening the idea that human RECQL5 interacts with actively transcribed yeast genes (Supplementary Fig. 6). RECQL5-enriched genes were significantly longer and highly expressed compared to the genome median values (Fig. 5C) with an over-representation of genes associated with ribosome biogenesis and RNA processing (Supplementary Table 5). Interestingly, RECQL5 was distributed in a 5′ → 3′ gradient comparable to that of the RNA elongation and processing factor Hpr1 but with a more pronounced peak at the 3′ end of the genes (Fig. 5D). These data are consistent with an RNAPII-driven transcription elongation and termination defect. RECQL5 was also recruited to tRNAs, introns, snRNAs, snoRNAs or centromeres and telomeres (Supplementary Table 4), implying an impact beyond its interaction with RNAPII, likely due to additional interactions with other proteins or the DNA itself.

## Discussion

To gain insight into the molecular mechanisms of human RECQL5 DNA helicase we expressed it in yeast. We found that high levels of RECQL5 in yeast cause growth inhibition and DNA damage, as determined by Rad52 foci accumulation. RECQL5 expressing cells are more sensitive to genotoxic agents and show a hyperrecombination phenotype that is intensified by active transcription. In addition, yeast cells expressing RECQL5 show a defect in Rad51 foci accumulation that is consistent with its role suppressing homologous recombination.

We found that the interaction between RNAPII and RECQL5 is conserved from yeast to humans (Fig. 4A). This interaction was also observed with the RECQL5-ID mutant that lacks the KIX domain, a motif necessary for binding with RNAPII non-phosphorylated (RNAPIIa) and hyperphosphorylated (RNAPIIo) (Aygun et al. 2009; Islam et al 2010). This result could be explained by the existence of another RNAPII interacting domain, SRI, that is able to mediate association with the RNAPIIo form. Indeed, both RECQL5 and RECQL5-ID bind to active chromatin as shown by ChIP analysis at the inducible *GAL1* gene (Fig. 4B).

Somewhat surprisingly, we found RECQL5-ID, a RECQL5 mutant that has previously been shown to perturb the association with RNAPII in vitro, still associated with the transcribing polymerase inside cells. Interestingly, the expression of both RECQL5 and RECQL5-ID lead to readthrough and recombination phenotypes, although in the case of mutant version the effect seems to be slightly lower. Given that different RECQL5 separation-of-function mutants of this kind have been used in previous studies (Aygun et al. 2009; Islam et al. 2010; Kanagaraj et al 2010), we suggest that the results from the use of such mutant be considered carefully if they do not directly show a loss of RNAPII interaction in cells as well. The scenario seems to be more complex than previously thought. Recently, an alternative isoform of human RECQL5 containing additional amino acids in the KIX domain has been shown to confer a different impact on transcription and genome instability than the ubiquitously expressed isoform, suggesting the existence of specialized isoforms in human cells (Ding et al. 2021).

Importantly, our data show a genome-wide recruitment of RECQL5 at actively transcribed regions that increases toward the 3′ end of genes (Fig. 5D). This result is consistent with previous analyses in human cells showing that the interaction of RECQL5 is dependent on the CTD phosphorylation of the elongating RNAPII (Kanagaraj et al. 2010). Human RECQL5 was shown to negatively affect transcription elongation, with increased elongation rates after helicase depletion and the opposite effect upon overexpression (Saponaro et al. 2014). A defect at transcription termination was detected at the highly expressed inducible genes *GAL10 and GAL1* (Fig. 4C; Supplementary Fig. 4). This observation was supported genome-wide by the accumulation of RECQL5 observed at the 3′ end of the genes in ChIP-chip analysis of yeast cells expressing RECQL5 (Fig. 5). These data suggest that an excess of this helicase could interfere in yeast with the recruitment of transcription termination factors.

Interestingly, microarray analysis of *tet::RECQL5* expressing cells show that down-regulated genes showed a higher GC content implying that the *RECQL5* expression could have a more pronounced impact at difficult-to-transcribe regions (Supplementary Fig. 6). It is possible that human RECQL5 expression in yeast negatively affected the function of the transcription elongation factor TFIIS, given the structural similarities between RECQL5 and TFIIS proteins and the reported competition of RECQL5 with the binding of TFIIS to RNAPII in human cells (Ding et al. 2021; Kassube et al. 2013). Supporting this hypothesis, we observed a slight effect of RECQL5 expression on *rpb1-S751F* (Supplementary Fig. 3), a mutant in a subunit of RNAPII in which slippage and transcription elongation defects as those of TFIIS have been reported (Strathern et al. 2013).

Our data suggest that expression of RECQL5 could be interfering with different transcription and processing factors. Interestingly, the heterologous expression of bacterial Rho factor, an RNA-dependent helicase/translocase in yeast is toxic and leads to aberrant transcripts as a consequence of an interference of this helicase with different mRNP biogenesis steps (Mosrin-Huaman et al 2009). In the case of RECQL5 further experiments are needed to see whether the transcription defects are also due to a disruption of RNA–protein interactions.

Expression of human RECQL5 in yeast confers a transcription-dependent genome instability phenotype (Figs. 2, 3), in agreement with previous reports in human cells indicating a role of RECQL5 in genome integrity associated with transcription stress (Saponaro et al. 2014). We show data suggesting that RECQL5 expression leads to R-loop dependent DNA damage (Fig. 3). These results suggest that an excess of this helicase could interfere with yeast machinery, transcription, and other helicases, promoting the formation of DNA-RNA hybrids. Indeed, RECQ functions have been linked to R-loop metabolism, thus in yeast, loss of yeast Sgs1 leads to R-loops, replication stalling and DNA damage (Chang et al 2017), and in human cells RECQL5 regulates TOP1 SUMOylation contributing to the reduction of R-loop formation (Li et al. 2015). The phenotype observed upon heterologous expression of RECQL5 in yeasts supports the conservation of this helicase during evolution. One hypothesis is that in *S. cerevisiae* RECQL5 functions could be carried out by different RecQ homologs, such Sgs1 and Hrq1, the two RECQ homologs described in *S. cerevisiae*, or even other helicases, as a safekeeper of genome integrity.

Our study indicates that as in human cells RECQL5 may function in association with RNAPII, having indeed a negative effect in transcription termination and genome stability. Therefore, analysis of the impact of RECQL5 heterologous expression in yeast, an organism lacking this protein, reveals new potential functional abilities of human RECQL5, which in yeast seems to be covered by other DNA helicases.

### Supplementary Information

Below is the link to the electronic supplementary material.

Supplementary file1 Effect of RECQL5 expression in WT cells (W303-1AR5). Streaks-outs of yeast strains carrying plasmids pYES3::RECQL5, pYES3::RECQL5-ID containing GAL::RECQL5, GAL:RECQL5-ID, respectively, or empty pYES3 (control) on selective SC medium with 2% glucose (non-induced conditions), 2%-galactose supplemented with 0.05% glucose or 2%-galactose. Streaks-outs of two different transformants are shown (T1, T2). Photographs were taken after 3 days of growth at 30ºC (PDF 5372 KB)

Supplementary file2 Rad51-YFP foci formation in wild-type strain (ML149-8A) transformed with the empty plasmid (pCM184), referred as Control, or plasmid with tet::RECQL5 (pCM184-RECQL5). Cells were cultured to exponential state and MMS (0.01%) was added and incubated for two additional hours. 0.5% of WT cells showed 2 foci (PDF 496 KB)

Supplementary file3 Viability assay of WT (W303-1A), rpb1-1 (WRP1-12A), rpb1S751F (WSR8-5A), rpb2-10 (WRP2) and rpb9∆ (WRP9-3C) strains transformed with the vector with GAL::RECQL5. Ten-fold serial dilutions of exponentially growing cultures plated in selective medium with the indicated concentrations of carbon source to regulate the expression of the RECQL5 protein. Photographs were taken after 3 days of growth at 30ºC (PDF 2601 KB)

Supplementary file4 A) Northern blot analysis of GAL10 mRNAs in WT strain carrying plasmids with GAL::RECQL5, GAL::RECQL5-ID or the corresponding empty plasmid (Control). B) Northern blot analysis of GAL1 mRNAs in WT strain transformed with GAL::RECQL5, GAL::RECQL5-ID or the corresponding empty plasmid (Control). Graphics represent the corresponding mRNA signal relative to rDNA. Average and SEM of n=2 (PDF 846 KB)

Supplementary file5 Structural and functional features analysis of up/down-regulated genes to the genome median in RECQL5 expressing yeast. Median values are shown and line represents the genome median. p≤0.05; **, p≤0.01; ***, p≤0.001; (Mann-Whitney’s U-test) (PDF 479 KB)

Supplementary file6 RECQL5, Rpb3 and Hpr1 distribution at the highly transcribed PDC1-TRX1, TEF1 and CDC19 loci, represented as signal log2 IP/SUP ratio. Blue (RECQL5-IP), orange (Rpb3-IP) and yellow (Hpr1-IP) histograms (online version) represent the statistically significant binding clusters (P < 0.01, minimum run >100 bp, maximum gap <250 bp). Genes and other features are represented according to the SGD (PDF 568 KB)

Supplementary file7 List of strains, plasmids, primers and antibodies used in this work (XLSX 19 KB)

Supplementary file8 (XLSX 181 KB)

## Data Availability

Strains and plasmids are available upon request. Supplementary Table 1 contains detailed description of strains, plasmids and antibodies used in this study. Gene expression data of RECQL5-expressing cells are available at Gene Expression Omnibus accession number (GSE180112).The ChIP-chip data can be accessed at Gene Expression Omnibus (GSE180107). Data generated and analysed during this study are included in supplementary information files: Supplementary Tables 2 and 3 (transcriptome analysis of REQL5-expressing yeast cells); Supplementary Tables 4 and 5 (ChIP-chip analysis). Supplementary Figs. 1–3.
