# Supplementary figures and images for: Expression of human RECQL5 in Saccharomyces cerevisiae causes transcription defects and transcription-associated genome instability
Source: Mol Genet Genomics. 2024 May 26;299(1):59. doi: 10.1007/s00438-024-02152-3 (PMC11128410; doi:10.1007/s00438-024-02152-3)

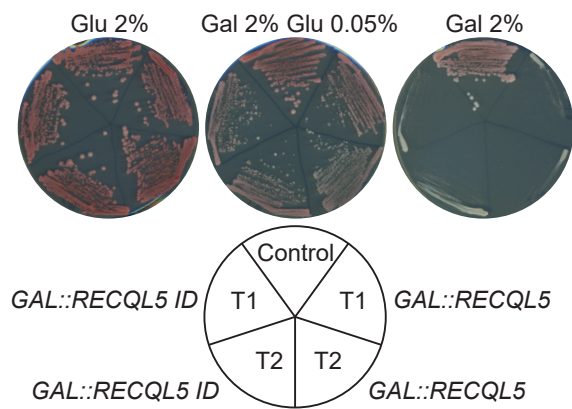

Supplement: Supplementary file 1 — Supplementary file1 Effect of RECQL5 expression in WT cells (W303-1AR5). Streaks-outs of yeast strains carrying plasmids pYES3::RECQL5, pYES3::RECQL5-ID containing GAL::RECQL5, GAL:RECQL5-ID, respectively, or empty pYES3 (control) on selective SC medium with 2% glucose (non-induced conditions), 2%-galactose supplemented with 0.05% glucose or 2%-galactose. Streaks-outs of two different transformants are shown (T1, T2). Photographs were taken after 3 days of growth at 30ºC (PDF 5372 KB) [file 438_2024_2152_MOESM1_ESM.pdf]

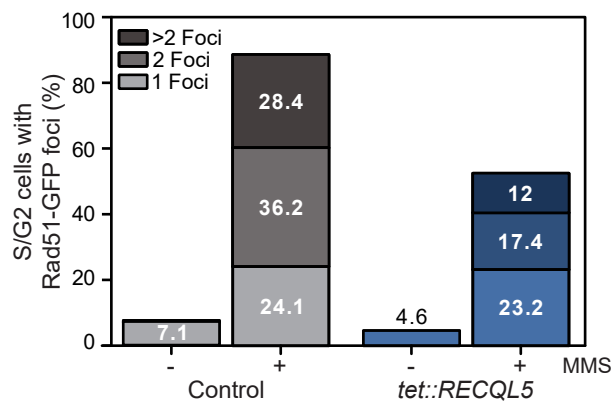

Supplement: Supplementary file 2 — Supplementary file2 Rad51-YFP foci formation in wild-type strain (ML149-8A) transformed with the empty plasmid (pCM184), referred as Control, or plasmid with tet::RECQL5 (pCM184-RECQL5). Cells were cultured to exponential state and MMS (0.01%) was added and incubated for two additional hours. 0.5% of WT cells showed 2 foci (PDF 496 KB) [file 438_2024_2152_MOESM2_ESM.pdf]

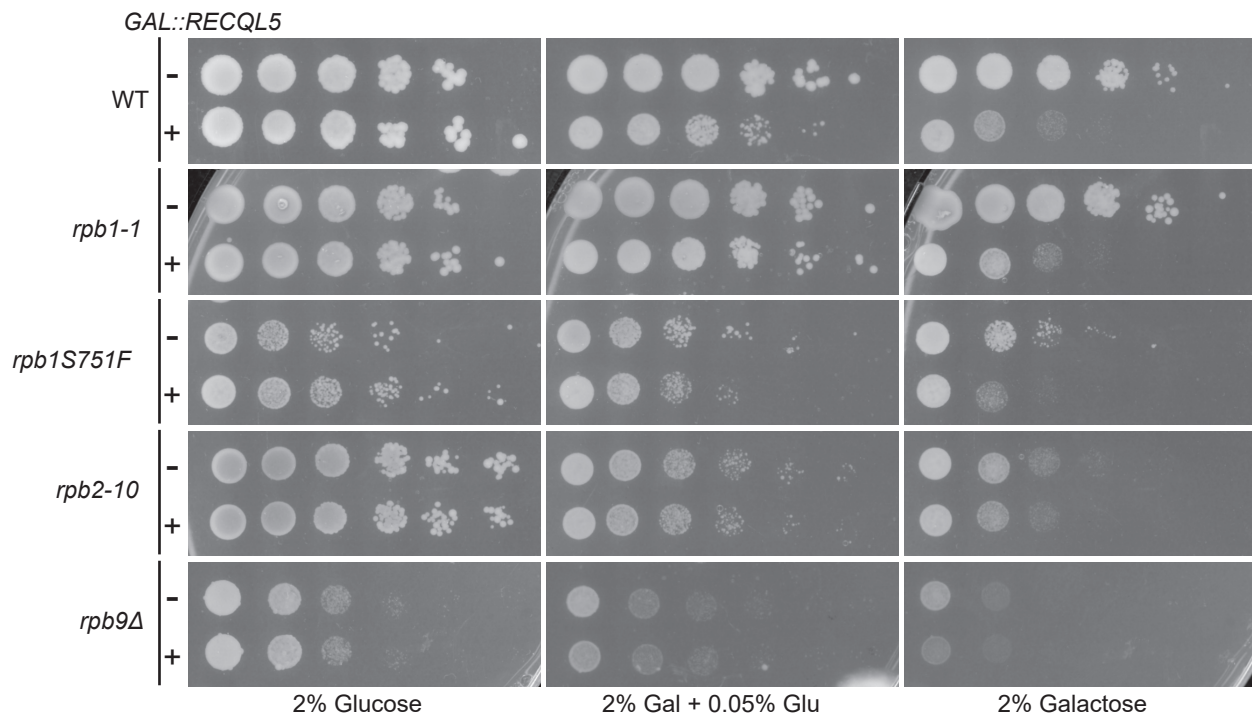

Supplement: Supplementary file 3 — Supplementary file3 Viability assay of WT (W303-1A), rpb1-1 (WRP1-12A), rpb1S751F (WSR8-5A), rpb2-10 (WRP2) and rpb9∆ (WRP9-3C) strains transformed with the vector with GAL::RECQL5. Ten-fold serial dilutions of exponentially growing cultures plated in selective medium with the indicated concentrations of carbon source to regulate the expression of the RECQL5 protein. Photographs were taken after 3 days of growth at 30ºC (PDF 2601 KB) [file 438_2024_2152_MOESM3_ESM.pdf]

**A**

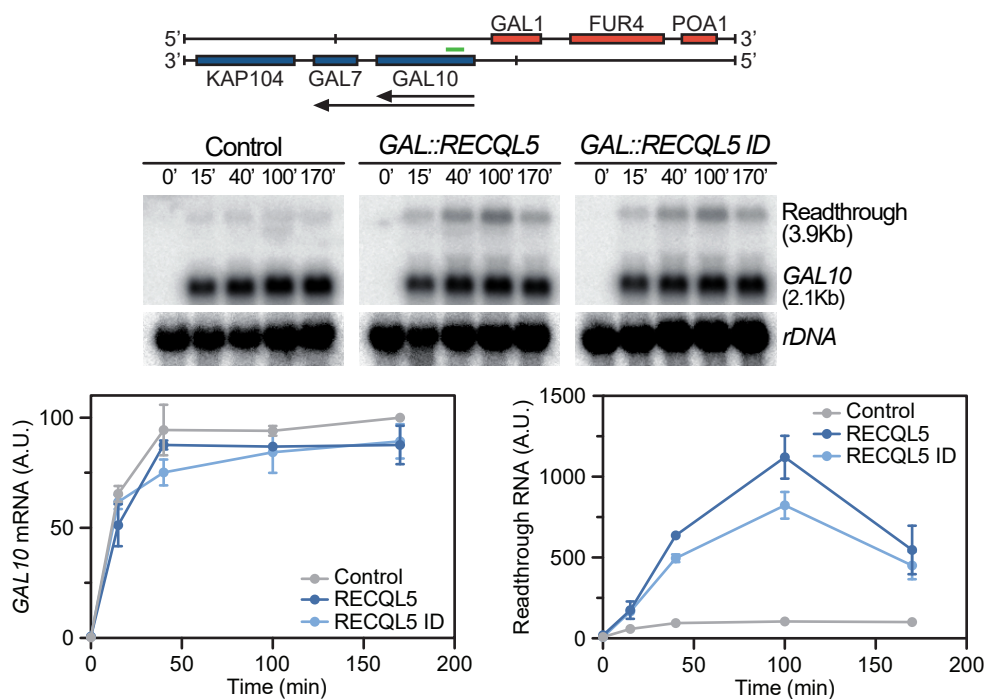

**B**

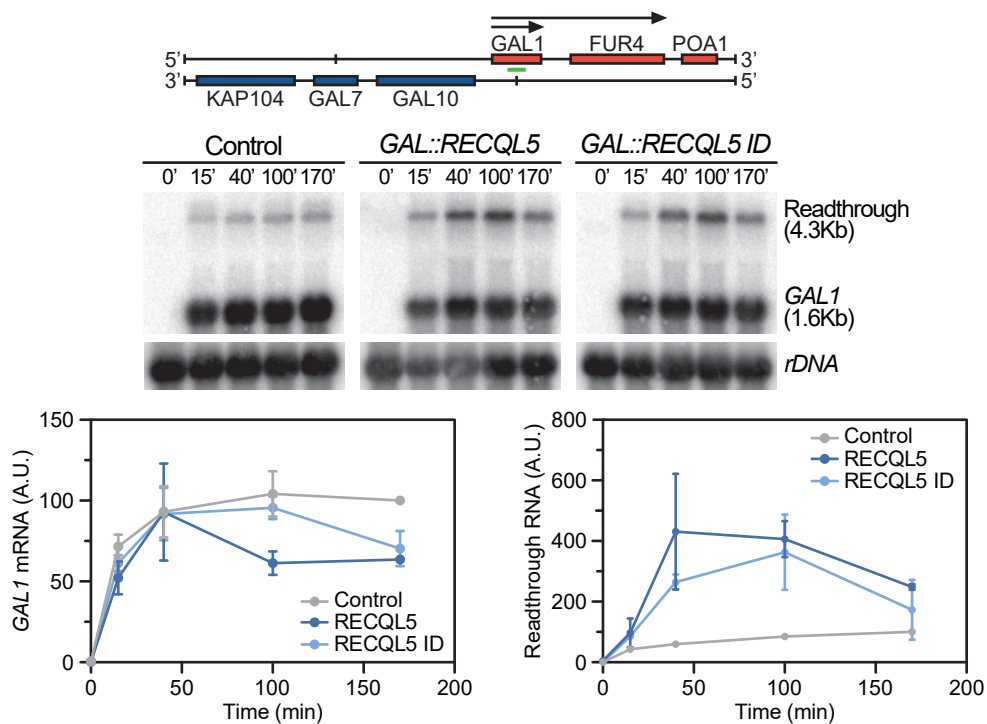

Supplement: Supplementary file 4 — Supplementary file4 A) Northern blot analysis of GAL10 mRNAs in WT strain carrying plasmids with GAL::RECQL5, GAL::RECQL5-ID or the corresponding empty plasmid (Control). B) Northern blot analysis of GAL1 mRNAs in WT strain transformed with GAL::RECQL5, GAL::RECQL5-ID or the corresponding empty plasmid (Control). Graphics represent the corresponding mRNA signal relative to rDNA. Average and SEM of n=2 (PDF 846 KB) [file 438_2024_2152_MOESM4_ESM.pdf]

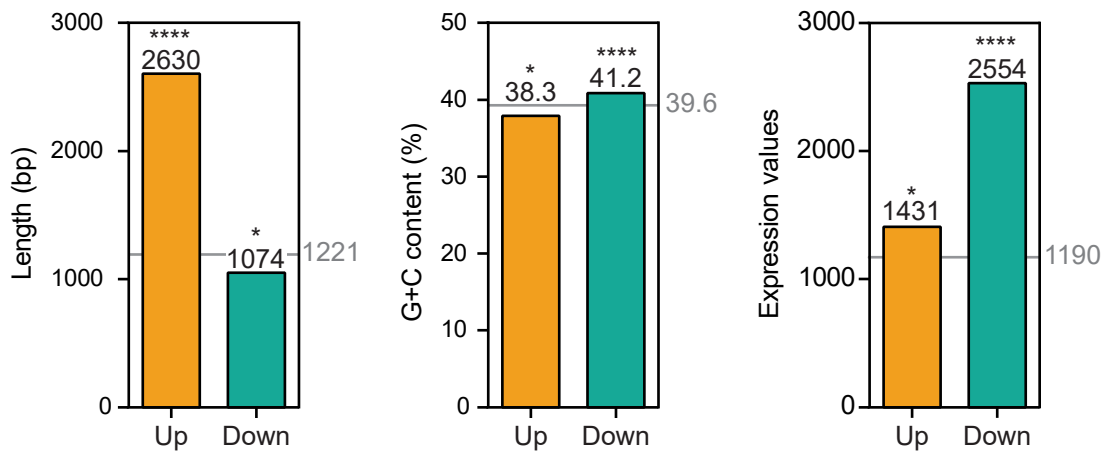

Supplement: Supplementary file 5 — Supplementary file5 Structural and functional features analysis of up/down-regulated genes to the genome median in RECQL5 expressing yeast. Median values are shown and line represents the genome median. p≤0.05; **, p≤0.01; ***, p≤0.001; (Mann-Whitney’s U-test) (PDF 479 KB) [file 438_2024_2152_MOESM5_ESM.pdf]

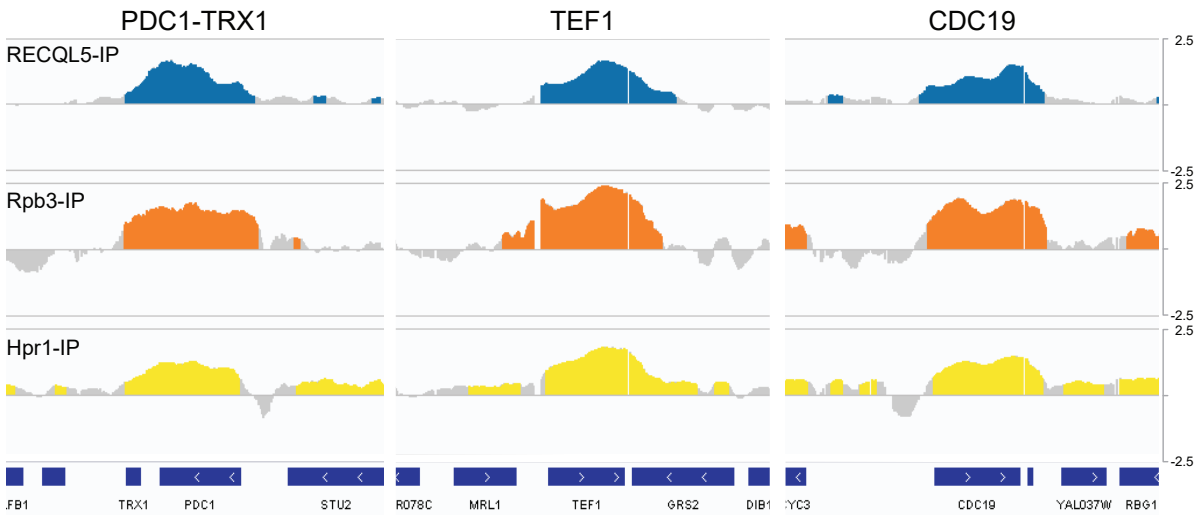

Supplement: Supplementary file 6 — Supplementary file6 RECQL5, Rpb3 and Hpr1 distribution at the highly transcribed PDC1-TRX1, TEF1 and CDC19 loci, represented as signal log2 IP/SUP ratio. Blue (RECQL5-IP), orange (Rpb3-IP) and yellow (Hpr1-IP) histograms (online version) represent the statistically significant binding clusters (P < 0.01, minimum run >100 bp, maximum gap <250 bp). Genes and other features are represented according to the SGD (PDF 568 KB) [file 438_2024_2152_MOESM6_ESM.pdf]
